# How interactions between ADHD and schools affect educational achievement: a family‐based genetically sensitive study

**DOI:** 10.1111/jcpp.13656

**Published:** 2022-07-04

**Authors:** Rosa Cheesman, Espen M. Eilertsen, Ziada Ayorech, Nicolai T. Borgen, Ole A. Andreassen, Henrik Larsson, Henrik Zachrisson, Fartein A. Torvik, Eivind Ystrom

**Affiliations:** ^1^ PROMENTA Research Center, Department of Psychology University of Oslo Oslo Norway; ^2^ Centre for Fertility and Health Norwegian Institute of Public Health Oslo Norway; ^3^ Department of Special Needs Education University of Oslo Oslo Norway; ^4^ NORMENT, Division of Mental Health and Addiction Oslo University Hospital Oslo Norway; ^5^ Institute of Clinical Medicine University of Oslo Oslo Norway; ^6^ Department of Medical Epidemiology and Biostatistics Karolinska Institutet Stockholm Sweden; ^7^ School of Medical Sciences Örebro University Örebro Sweden; ^8^ Department of Mental Disorders Norwegian Institute of Public Health Oslo Norway

**Keywords:** ADHD, gene–environment interaction, school performance, school, genetics

## Abstract

**Background:**

Children with ADHD tend to achieve less than their peers in school. It is unknown whether schools moderate this association. Nonrandom selection of children into schools related to variations in their ADHD risk poses a methodological problem.

**Methods:**

We linked data on ADHD symptoms of inattention and hyperactivity and parent–child ADHD polygenic scores (PGS) from the Norwegian Mother, Father, and Child Cohort Study (MoBa) to achievement in standardised tests and school identifiers. We estimated interactions of schools with individual differences between students in inattention, hyperactivity, and ADHD‐PGS using multilevel models with random slopes for ADHD effects on achievement over schools. In our PGS analyses, we adjust for parental selection of schools by adjusting for parental ADHD‐PGS (a within‐family PGS design). We then tested whether five school sociodemographic measures explained any interactions.

**Results:**

Analysis of up to 23,598 students attending 2,579 schools revealed interactions between school and ADHD effects on achievement. The variability between schools in the effects of inattention, hyperactivity and within‐family ADHD‐PGS on achievement was 0.08, 0.07 and 0.05 SDs, respectively. For example, the average effect of inattention on achievement was β = −0.23 (*SE* = 0.009), but in 2.5% of schools with the weakest effects, the value was −0.07 or less. ADHD has a weaker effect on achievement in higher‐performing schools. Schools make more of a difference to the achievements of students with higher levels of ADHD, explaining over four times as much variance in achievement for those with high versus average inattention symptoms. School sociodemographic measures could not explain the ADHD‐by‐school interactions.

**Conclusions:**

Although ADHD symptoms and genetic risk tend to hinder achievement, schools where their effects are weaker do exist. Differences between schools in support for children with ADHD should be evened out.

## Introduction

Complex outcomes such as educational achievement result from exchanges between individual characteristics and environmental contexts such as families, schools and neighbourhoods, over time (Bronfenbrenner & Ceci, 1994). Schools are essential for cognitive and social development and have been proposed to affect student achievement in multiple ways, including through social processes (e.g. quality of instruction, disciplinary practices) and physical and institutional features (e.g. school resources, class sizes, ability tracking) (Rutter, 1982; Wang & Degol, 2016). However, it is not well understood how school environments interact with individual differences between children in their cognitive and behavioural difficulties. Research in this area could eventually inform policymakers and teachers on which school environments best support children at risk of low achievement.

Attention‐deficit/hyperactivity disorder (ADHD) is of particular interest in this context. Its characteristic symptoms of hyperactivity and inattention loom large at school. ADHD symptoms are common, typically appear in early childhood and are associated with profound impairments in achievement (DuPaul et al., 2004; Massetti et al., 2008; Polderman, Boomsma, Bartels, Verhulst, & Huizink, 2010; Thapar & Rutter, 2015). Academic impairments are especially severe for inattention symptoms (Pingault et al., 2011; Salla et al., 2016) and may be partly due to general effects on everyday learning, such as spending more time off‐task than peers (Kofler, Rapport, & Alderson, 2008). However, the role of schools in the relationship between ADHD and academic difficulties has received relatively little attention. School‐based behavioural interventions (e.g. environmental modifications, reinforcement systems, computer‐assisted instruction and peer tutoring) improve behaviour but have modest effects on achievement (DuPaul, Weyandt, & Janusis, 2011; Jangmo et al., 2019). More research is required on school factors that directly address academic skills in ADHD (DuPaul et al., 2011). Identifying which school environments aid the achievements of students with ADHD could help to design policies and interventions.

Quantifying how much the association between ADHD and achievement varies between schools is a valuable first step towards understanding how schools influence the academic consequences of ADHD. Addressing this question using a latent approach opens the possibility of capturing effects of all school‐level factors driving differences in how much ADHD symptoms affect achievement, including unknown and unmeasured ones. This has the advantage of remaining agnostic to specific environments, which are challenging to identify and measure and, if measured, may represent mere proxies for the ‘true’ interactive school factors (Boardman, Daw, & Freese, 2013; Trejo et al., 2018). After clarifying whether school environments moderate ADHD effects on achievement (i.e. increase or decrease the effect compared with the average across schools), researchers may then identify what these environments are. If available measured school factors cannot account for the latent moderation effects, this would justify hypothesising about other specific school characteristics.

ADHD‐by‐school interactions are challenging to study because non‐random selection into schools can create spurious interactions. For example, if parents with elevated ADHD symptoms choose certain schools for their offspring, then the interaction between student ADHD symptoms and schools may actually reflect an interplay between student and parent symptoms. This endogeneity problem can be tackled through within‐family analysis of children's ADHD polygenic scores (PGS) adjusted for parents' ADHD‐PGS. ADHD is strongly genetically influenced (70–80% in twin studies) (Faraone & Larsson, 2019), and PGS allow genetic propensity for ADHD to be indexed on the individual level. Parents' selection of schools partly reflects heritable characteristics such as ADHD liability, which are inherited by children. This mechanism, known as *passive gene–environment correlation* (Plomin, DeFries, & Loehlin, 1977), leads to a correlation between child genetic variation and the school they attend. Importantly, school selection by parents confounds typical PGS analyses, but not within‐family analyses where the child PGS is conditioned on parental PGS (Schmitz & Conley, 2017). Within‐family PGS effects reflect the random segregation of alleles at conception and are uncorrelated with the social environments that parents select. However, in situations where children can select schools themselves, the within‐family PGS may still be correlated with school (i.e. active gene–environment correlation is not adjusted for). Previous work has demonstrated negative effects of the ADHD‐PGS on achievement (Greven, Kovas, Willcutt, Petrill, & Plomin, 2014; Stergiakouli et al., 2017), including within families (Jangmo et al., 2021), but no research has examined between‐school differences in this association.

Between‐school variation in the effect of ADHD genetic risk on educational achievement would constitute a gene–environment interaction; that is, genes and social environments moderate one another (Plomin et al., 1977). In both the educational achievement and ADHD literatures, empirical findings on gene–environment interactions have been mixed, with genetic influences being stronger, weaker or invariable between environments. Studies have focused on interactions with specific measured home environments, such as family socioeconomic status (Figlio, Freese, Karbownik, & Roth, 2017; Gould, Coventry, Olson, & Byrne, 2018; Pennington et al., 2009; Tucker‐Drob & Bates, 2016), family chaos (Gould et al., 2018; Z. Wang, Deater‐Deckard, Petrill, & Thompson, 2012), parental conflict and involvement (M. A. Nikolas, Klump, & Burt, 2015; M. Nikolas, Klump, & Burt, 2012). A few studies have examined gene–environment interactions involving schools, finding that genetic effects on oral reading fluency in second grade increased as teacher quality increased (Taylor, Roehrig, Soden Hensler, Connor, & Schatschneider, 2010), but that associations between the educational attainment PGS and adult educational and socioeconomic attainments generally do not meaningfully vary between schools (Trejo et al., 2018). However, these have been United States‐based and have not considered the role of ADHD or its genetic risk.

Here, we explore how educational achievement is shaped by interactions between individual differences in ADHD and school environments. Our primary aim was to estimate total latent between‐school variation in the effects of inattention, hyperactivity and within‐family ADHD‐PGS on achievement. Using multilevel models, we remain agnostic to the relevant aspects of school environments. Moreover, our within‐family PGS analyses control for selection into schools. Our data set includes >25,000 Norwegian children's national test results, school identifiers, ADHD symptoms and parent–offspring PGS for ADHD, from a novel linkage between genetic trio data in the Norwegian Mother, Father and Child Cohort Study (Magnus et al., 2016) and the Norwegian National Educational Database. Norway is a useful context because the education system is highly standardised, and register data are almost complete. In sum, these data uniquely position us to investigate how genetic propensity interacts with schools in explaining the negative association between ADHD and achievement.

## Methods

### The Norwegian context

Relative to other Western nations, Norway is a wealthy welfare state with low unemployment and low economic inequality (Eurofound, 2017). Nonetheless, income inequality and child poverty are substantial, and exacerbating over time (Barth, Moene, & Pedersen, 2021). The public sector provides various welfare services, including free compulsory education and universal health care. Education is comprehensive with a common curriculum for all students, and there is no tracking/streaming before upper secondary schooling. In elementary school, children primarily attend local schools, defined by their home address. Fewer than 4% of students attend private schools, which are schools with alternative pedagogical traditions, religious schools, or international schools. Special needs schools are extremely rare: in 2018, only 0.65% of children attended such schools. Child mental health services are almost exclusively public, and children with suspected ADHD or other mental health problems are referred by general practitioners. National guidelines for treatment of ADHD refer to adapted and special needs education as a key intervention, either prioritised before, or supplementary to psychopharmacological treatment. Local school psychology services design individual pedagogical plans based on symptoms and child functioning, not on diagnoses (Helsedirektoratet, 2021).

### Sample

The Norwegian Mother, Father, and Child Cohort Study (MoBa; Magnus et al., 2016) is a prospective population‐based pregnancy cohort study conducted by the Norwegian Institute of Public Health. Pregnant women were recruited from across Norway from 1999 to 2009. The women consented to initial participation in 41% of the pregnancies. The total cohort includes 114,500 children, 95,200 mothers and 75,200 fathers. All initial MoBa participants are currently being genotyped, and the sample of 98,110 genotyped individuals available to date should not be systematically different to MoBa overall. The current study is based on version 12 of the quality‐assured phenotype data files.

The present analyses were performed on a subsample of ~23,000 MoBa children with genome‐wide genotype data, parental genotype data, plus administrative records of educational achievement and school membership linked to MoBa through the Norwegian national ID number system. The administrative data are of high quality and do not suffer from attrition (Hovde Lyngstad & Skardhamar, 2011; Røed & Raaum, 2003). Analyses were restricted to one child per family, by choosing one sibling at random. Phenotypic‐only analyses were additionally limited to ~11,000 MoBa children with data for mother‐rated ADHD symptoms at age 8. This lower sample size is mainly due to attrition as the symptom data were collected when children were 8 years of age, whereas the genotype data were collected at birth. See Appendix S1 for a sample size flow chart.

### Ethics

The establishment of MoBa and initial data collection was based on a licence from the Norwegian Data Protection Agency and approval from the Regional Committees for Medical and Health Research Ethics. The MoBa cohort is now based on regulations related to the Norwegian Health Registry Act. The current study was approved by the Regional Committees for Medical and Health Research Ethics (project #2017/2205).

### Measures

#### School achievement

Standardised national test results for maths and reading at grades 5, 8 and 9 (ages 10, 13 and 14), and English at grades 5 and 8 were obtained through linkage to Norway's National Education Database. Introduced in 2007, these tests are mainly used to monitor school development over time. Tests are compulsory, with 96% of all students in Norway taking them; students with special needs and those following introductory language courses may be exempt. Results are conveyed to teachers and parents but have no direct consequence for students. We residualised students' test scores for sex, current age (to capture birth cohort effects) and the exact age when they took the tests. We created ‘core achievement’ measures as mean scores at each grade across available subjects (Rimfeld et al., 2018), and centred these to have mean zero and standard deviation 1. Results are presented for overall mean achievement in the main text.

Interactions can arise spuriously due to features of outcome distributions (Domingue, Trejo, Armstrong‐Carter, & Tucker‐Drob, 2020), for example a truncated distribution of educational attainment (Trejo et al., 2018). To guard against this, we assessed the mean, variance and skewness of test outcome distributions (overall and stratified by school performance deciles) and investigated their correlations with item response theory (IRT) scores. The IRT scores, which measure latent ability, are more normally distributed but less contextually meaningful than the main standardised test outcomes returned to teachers.

#### School codes

We matched children's achievement results to the schools they attended when they took each test. School identifiers were obtained from the National Education Database.

#### School sociodemographics

To complement the latent analyses, we tested whether specific school‐level sociodemographic measures could explain interactions identified through multilevel modelling. We created sociodemographic measures by aggregating administrative data from all parents of students at each school with register data, not only MoBa participants. Measures were intended to capture both the average sociodemographic background among students within each school and the variability of sociodemographic backgrounds of students within each school. For each school, we included five measures. The first measure was the average years of completed education of parents, converted from Norwegian Standard Classification of Education (NUS2000) categories and measured when students were 16. The second sociodemographic indicator was the average parental pretax annual income from gainful employment including self‐employment but not capital income or social welfare transfers. We averaged the income of both parents across the years that children were aged 11–15, and ranked their income compared with other parents in the same birth cohort. Third and fourth, we measured socioeconomic inequality by calculating Gini coefficients in reported levels of parental education and income, respectively. Gini is a widely used single measure of inequality and ranges from 0 to 1, with 0 indicating absolute equality and 1 indicating absolute inequality. Fifth, we calculated the proportion of children who are non‐Western immigrants and/or who are the children of non‐Western immigrants. We created these broad measures in the absence of more detailed school data. Notably, the measures could capture effects intrinsic to specific schools (e.g. peer effects) or broader social stratification (e.g. composition of the school catchment area). If the latter is true, then these variables could be considered additional controls for selection into schools and neighbourhoods.

We used the same measures of parental educational attainment and earned income as individual‐level control variables to ensure that interactions are not confounded by differences in family socioeconomic background.

#### ADHD symptoms

The Parent Rating Scale for Disruptive Behaviour Disorders (RS‐DBD) (Silva et al., 2005) was used to measure inattention and hyperactivity symptoms when children were aged 8. Items were rated by mothers on a 4‐point scale (1 = never/rarely, 2 = sometimes, 3 = often and 4 = very often). Inattention items (9 in total) included ‘Fails to give close attention to details or makes careless mistakes in schoolwork’ and ‘Avoids, dislikes or is reluctant to engage in tasks that require sustained mental effort (such as schoolwork or homework)’. Hyperactivity items (9 in total) included ‘Leaves seat in classroom or in other situations in which remaining seated is expected’ and ‘Talks excessively’. Item alphas ranged from 0.78 to 0.96 (https://www.fhi.no/en/studies/moba/for‐forskere‐artikler/questionnaires‐from‐moba/). We summed the items for the inattention and hyperactivity subscales. For individuals with less than 50% missing data on either scale, missing item values were imputed by the sample mean. The remaining individuals were excluded from analyses. We then centred sum scores to have mean zero and standard deviation 1. Inattention and hyperactivity subscales were analysed separately, due to differences in their aetiologies (Nikolas & Burt, 2010) and associations with achievement (Salla et al., 2016). These symptom measures are intended to capture population variation in ADHD as a dimensional trait, of which clinical ADHD is the extreme (Faraone & Larsson, 2019). Findings based on symptoms rather than diagnoses are more generalisable and robust to variability in diagnostic practices.

### Genotype quality control

The current MoBa genomic data set comprises imputed genetic data for 98,110 individuals (~32,000 parent–offspring trios; before quality control), derived from nine batches of participants, who make up four study cohorts. Within each batch, parent and offspring genetic data were quality‐controlled separately. Preimputation quality control criteria have been described in previous publications and are detailed in the Appendix S2. We conducted postimputation quality control, retaining SNPs meeting the following criteria: imputation quality score ≥0.8 in all batches, nonduplicated (by position or name), call rate > 98%, minor allele frequency >1%, Hardy–Weinberg equilibrium *p* < .001, not associated with genotyping batch at the genome‐wide level and not causing a Mendelian error. We removed individuals with the following criteria: heterozygosity outliers (F‐het ±0.2), call rate < 98%, reported sex mismatching SNP‐based sex, duplicates (identified using PLINK's (Chang et al., 2015) ‐genome command as having pihat > = 0.98, and distinguished from monozygotic twins through linkage to unique IDs in the population register, plus age, sex and kinship information within MoBa), individuals with excessive numbers of close relatives (cryptic relatedness) and Mendelian errors. To minimise environmental confounding, we identified a subsample of individuals with European ancestries via principal component analysis using the 1,000 Genomes reference; thresholds for exclusion of outliers were based on visual inspection of a plot of principal components 1 and 2. The final numbers of individuals and SNPs passing quality control were 93,582 and 6,797,215, respectively. Principal components of genetic ancestry were computed for all participants using PLINK's ‐within and ‐pca‐clusters commands, based on an LD‐pruned version of the final quality‐controlled genotype data.

### ADHD polygenic scores (PGS)

We generated ADHD‐PGS for all individuals in MoBa who passed quality control, based on the latest genome‐wide association study of 20,183 individuals of European‐associated ancestry diagnosed with ADHD and 35,191 controls (Demontis et al., 2019). We used the PRSice software to calculate scores using all SNPs (i.e. *p*‐value threshold of 1), with clumping parameters kb = 500, *p* = 1, *r* ^2^ = .25 (Choi Shing Wan, n.d.). We excluded 175 MoBa participants who were also included in the BUPGEN and TOP cohorts contributing to the ADHD‐GWAS prior to analyses. We computed mid‐parental PGS by taking the average maternal and paternal PGS. ADHD‐PGS for one child per family (*N* = 27,582; one sibling selected randomly) and mid‐parental PGS (*N* = 25,169, hereafter ‘parental PGS’) were then centred to have mean zero and standard deviation 1. In all PGS analyses, we included parental PGS as controls, such that effects of offspring PGS are *within‐family direct genetic effects*. These within‐family PGS effects reflect random genetic segregation, holding any parental and wider ancestral differences constant, including population stratification, assortative mating and general social background effects. Notably, within‐family designs based on siblings or adoptees rather than parent–offspring trios can also be used to distinguish direct genetic effects from parental genetic effects (Demange et al., 2020). We also included principal components (5 based on maternal data and 5 based on paternal data) to control for population stratification in the parental ADHD‐PGS effects.

The main advantage of the within‐family ADHD‐PGS over the symptom measures is that it effectively accounts for differential selection of schools by parents. We checked the clustering of children in schools according to ADHD symptoms and PGS. For inattention and hyperactivity symptoms, and individual child and parental ADHD‐PGS, 1–2% of the variance is at the school level, which indicates selection into schools (Table S1). However, once parental ADHD‐PGS is accounted for, 0% of the variance in child ADHD‐PGS is explained by schools. The absence of clustering of child ADHD at the school level in a within‐family ADHD‐PGS model implies that we can consider the sorting of students into schools according to within‐family ADHD‐PGS in this model as random and interpret the variation between schools in PGS effects causally. Note that the degree of clustering of genetic risk for ADHD in schools is likely to be larger than estimated here using child and parent PGS, which only explain 1.4% and 0.6% of the variance in child ADHD symptoms, respectively (effects were equal for inattention and hyperactivity subscales).

### Statistical analyses

Table 1 summarises our approach to investigating ADHD‐by‐school interactions. In short, we estimated the effects of ADHD on achievement in standardised tests and examined whether these associations varied between schools. We performed analyses for three indices of ADHD (inattention, hyperactivity and within‐family ADHD‐PGS) separately. To ensure that any between‐school variability was not simply produced by chance, we formally compared fit statistics for a series of increasingly complex multilevel models (see below).

**Table 1:** Model‐fitting approach

Model	Fixed effects	Random effects
1. Base	ADHD Grade	Individual child
2. Between‐school outcome differences	ADHD Grade	Individual child School
3. ADHD‐by‐school interaction	ADHD Grade	Individual child ADHD|School
4. ADHD‐by‐school interaction, adjusted for parental socioeconomic status	ADHD Grade Parental income Parental education	Individual child ADHD|School
5. Accounting for school outcome differences	ADHD Grade Parental income Parental education School‐average parental income School‐average parental education School inequality in parental income School inequality in parental education School proportion of non‐Western immigrants	Individual child ADHD|School
6. Accounting for ADHD‐by‐school interaction	ADHD Grade Parental income Parental education School‐average parental income School‐average parental education School inequality in parental income School inequality in parental education School proportion of non‐Western immigrants School‐average parental income*ADHD School‐average parental education*ADHD School inequality in parental income*ADHD School inequality in parental education*ADHD School proportion of non‐Western immigrants*ADHD	Individual child ADHD|School

The dependent variable was educational achievement (standardised national test results); ADHD refers to inattention symptoms, hyperactivity symptoms or the within‐family ADHD‐PGS – we performed the model comparison procedure separately for these three ADHD indicators; ADHD|School refers to the random slope for the ADHD effect between schools (consistent with the lme4 R package notation).

The base model (Model 1) estimated the association between achievement and ADHD (symptoms or within‐family PGS). We pooled data across grades by including individual identification number as a random intercept, and time point as a fixed effect to account for mean differences in scores across time. Time point was coded as a continuous variable centred with 0 for grade 9, −1 for grade 8 and − 4 for grade 5. Note that the grade 9 composite only includes maths and reading, whereas achievement composites for grades 5 and 8 include all three subjects.

In Model 2, we tested the degree to which achievement varied between schools, by adding a random intercept for schools.

In Model 3, we tested for ADHD‐by‐school interactions by adding random slopes for the effects of ADHD on achievement across schools. Improved fit relative to Model 2 is evidence that there is more variation around the average effect of ADHD (or *slope)* between schools than expected by chance. Since random intercepts were already included in Model 2, improved fit of Model 3 must only reflect schools interacting with ADHD, and not mean differences between schools.

In Model 4, we aimed to correct the school‐specific intercepts for potential selection into schools by adding fixed effects for individual‐level parental educational attainment and income. Intercepts thus reflect school effects on achievement rather than differences between students in family socioeconomic background.

We also tested whether school‐level sociodemographic characteristics could explain any observed school effects and ADHD‐by‐school interactions. We first added fixed effects for school environmental measures (Model 5). These fixed effects are meant to account for intercept variance, that is average differences between schools in student achievement. We then added environment‐by‐ADHD interaction terms (Model 6) to see whether the covariates could explain ADHD–school interactions, beyond any main effects that they have on achievement (already accounted for in Model 5). The five school sociodemographic measures were tested jointly. If covariates account for interactions between ADHD and schools, then the fit of Model 6 would be improved relative to Model 5, and the standard deviation of slopes for ADHD measures between schools would be reduced.

Since we used maximum‐likelihood estimation, we could compare fit of models before and after the inclusion of fixed effects, not only of models that differ in their random‐effects structure. Specifically, this allowed us to test whether model fit improved upon inclusion of random slopes for ADHD effects across schools (i.e. an ADHD–school interaction) and upon inclusion of specific school variables as fixed effects (e.g. school‐average parental income) that could explain any interactions.

Multilevel models allow the inclusion of all schools in analyses, even those only attended by one individual. Although having larger numbers of individuals per group increases power (Austin & Leckie, 2018), small/singleton groups do not lead to bias, because the models borrow information from other groups (Bell, Ferron, & Kromrey, 2008). By including the single‐student schools, our results become more population‐representative. In any case, the single‐student schools generally have not one but three outcome observations (for grades 5, 8 and 9), and the number of schools (of which we have many: ~2,500) is more important for statistical power than the number of children per school.

We performed two supplementary analyses. First, we tested for gender differences in any ADHD‐by‐school interactions by refitting the best model for boys and girls separately. Second, we explored whether results could be partly due to between‐school differences in special education services. We used parent‐reported information from MoBa (at child age 8) on whether a formal administrative decision had been made about their child being eligible for special education. By comparing the fit of further multilevel models, we tested whether the effect of ADHD on achievement depended on receiving special educational support, and whether this interaction varied between schools (see Appendix S3 for full details).

#### Model fitting and comparisons

Analyses were conducted in R with the lme4 package (Bates, Mächler, Bolker, & Walker, 2015). Model comparisons are made using the anova() command to assess AIC fit statistics. AIC calculates the trade‐off between model fit and model complexity with a penalty for the number of parameters. If the model with, for instance, the random slopes (ADHD effects) across schools has a lower AIC value than that of a simpler model, this is evidence that ADHD‐by‐school interactions should be included for an optimal approximation of the underlying data generating processes.

## Results

### Descriptive statistics

Achievement outcomes (standardised national test scores) were approximately normally distributed, with no indication of skewness of <−1 or >1 or of ceiling effects (no students gained the maximum number of points; Table S2). This held across deciles of school quality (Figures S1–S3). Additionally, achievement outcomes were strongly correlated with item response theory‐derived scores reflecting the latent ability distribution, from the Norwegian Education database (Figure S4). The ADHD symptom measures were correlated at 0.62, and the within‐family ADHD‐PGS was equally correlated with inattention and hyperactivity (0.03 and 0.04, respectively).

Multilevel models included up to 23,598 children with complete data for achievement, ADHD‐PGS, parental ADHD‐PGS, and school membership (2,579 unique schools). The average number of students participating in MoBagenetics per school at grade 5 was 11, with a minimum of 1 (in 221 schools) and a maximum of 65 (in one school). For the analyses with ADHD symptoms, the sample included 11,737 individuals, from 2,383 schools. Table S2 shows descriptive statistics for all variables.

### ADHD‐by‐school interactions influence educational achievement

To explore between‐school differences in the effects of ADHD on achievement, we compared models as outlined in Table 1. Model‐fitting results showed that the best model for all three ADHD indicators (inattention symptoms, hyperactivity symptoms and within‐family ADHD‐PGS) was Model 4. Table 2 shows that AIC values were lowest for this model, in which ADHD effects on achievement were allowed to vary between schools (i.e. an ADHD‐by‐school interaction), and parental education and income were controlled for. In all cases, the change in AIC indicated substantial improvement in fit upon inclusion of the ADHD–school interaction (change in AIC between Models 2 and 3 was 74, 59 and 11 for inattention, hyperactivity and within‐family ADHD‐PGS, respectively). Table S3 shows full model fit statistics (chi‐squared, BIC, log‐likelihood, etc., as well as AIC). For Model 3 compared to Model 2, chi‐squared *p*‐values were <2e−16 for inattention and hyperactivity, and 0.001 for the within‐family ADHD‐PGS. For Model 4 compared with Model 3 (i.e. the addition of controls for parental socioeconomic status), chi‐squared *p*‐values were <2e−16 for all three ADHD indicators. We now describe the estimates for the best‐fitting model (Model 4). Full results for all tested models are in Table S4.

**Table 2:** AIC fit statistics for tested models, with the best‐fitting models in bold

Model	ADHD variable		
	Inattention	Hyperactivity	Within‐family ADHD‐PGS
1. Base	49,500	50,164	99,445
2. Between‐school outcome differences	49,195	49,879	98,515
3. ADHD‐by‐school interaction	49,121	49,820	98,504
4. ADHD‐by‐school interaction (lower N with extra covariates)	44,338	44,969	97,083
5. ADHD‐by‐school interaction, adjusted for parent SES	**43,439**	**44,035**	**94,769**
6. Accounting for school outcome differences	**43,324**	**43,945**	**94,588**
7. Accounting for ADHD‐by‐school interaction	43,330	43,947	94,591

Note that model fitting was performed in two stages, first to test for the presence of ADHD‐by‐school interaction, and second to account for any interaction using school‐level variables.

**Figure 1:**
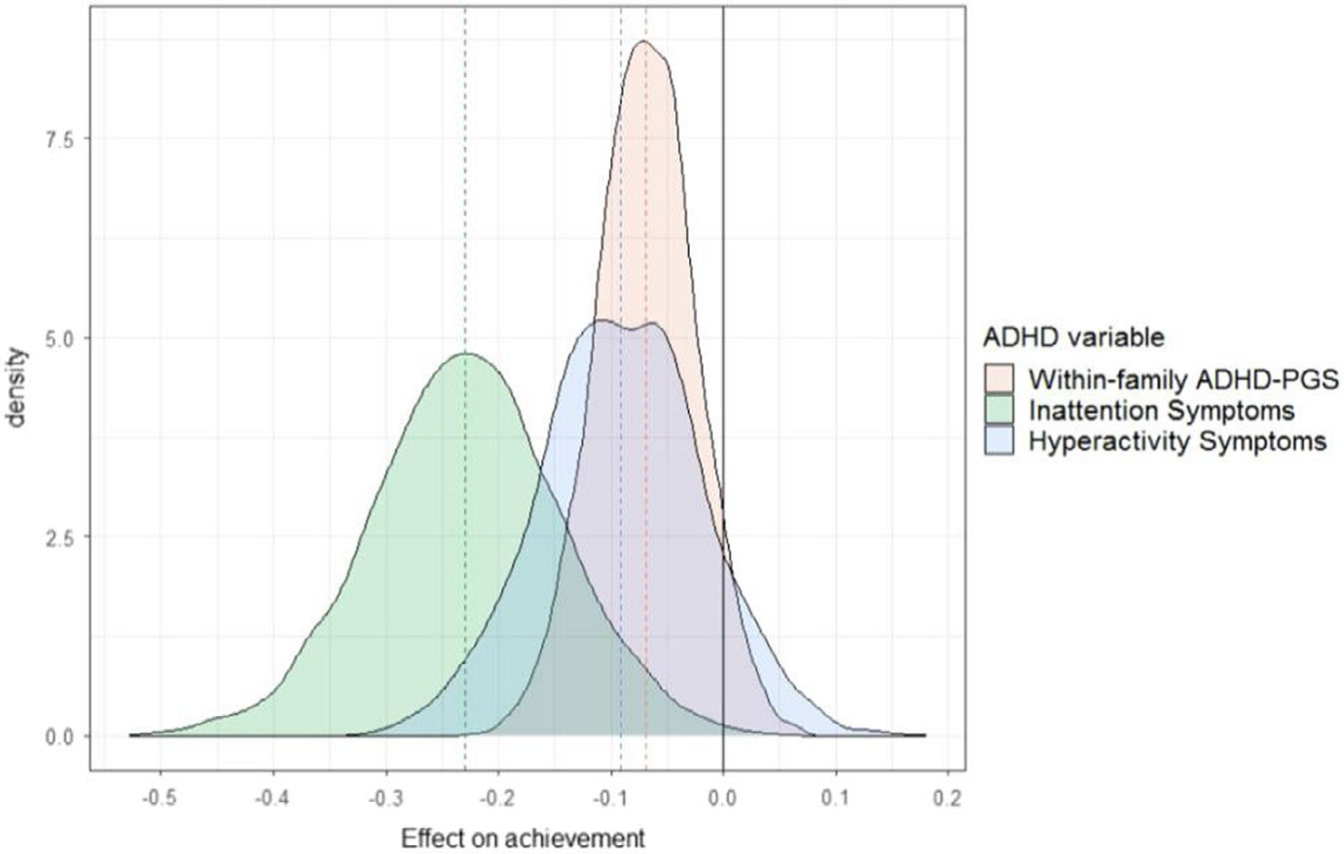
School‐level variation in the effect of ADHD measures on achievement. Dashed lines show the average effects of ADHD measures on achievement (β = −.23, −.09 and −.07 for inattention, hyperactivity and within‐family ADHD‐PGS, respectively). Normal curves show that there is variation in these associations between schools, with SDs of the distributions of effects across schools of 0.08, 0.07, and 0.05 for inattention, hyperactivity and within‐family ADHD‐PGS, respectively. Results for the three ADHD variables were obtained from three separate models [Colour figure can be viewed at wileyonlinelibrary.com]

Figure 1 displays results from the best‐fitting model for each ADHD indicator (Model 4), showing that the effects of all three on achievement (*x*‐axis) vary considerably between schools. On average (dashed lines), the association between inattention and achievement is β = .23 (*SE* = 0.009), while the association between hyperactivity and achievement is β = .09 (*SE* = 0.009). For children's within‐family ADHD‐PGS, the association with achievement is β = .07 (*SE* = 0.013). These effects varied between schools (indicated by the distribution of effects around the dashed averages in Figure 1), with standard deviations of effects between schools of 0.08, 0.07 and 0.05 for inattention, hyperactivity and within‐family ADHD‐PGS, respectively. This means that students' inattention symptoms have an effect between β = −.39 and β = −.07 in 95% of schools, but effects are more extreme than this in 5% of schools (calculated as −0.23 −/+ (1.96 × 0.08), since effects are normally distributed across schools).

The effect of the within‐family ADHD‐PGS (the child ADHD‐PGS effect adjusted for the parent ADHD‐PGS effect) on achievement is more extreme than double the school‐average effect (i.e. β = −.16) in 2.5% of the schools with the most strongly negative slopes, while negligible for schools in the opposite tail of the distribution of effects. Between‐school differences in within‐family ADHD‐PGS effects reflect gene–environment interaction, controlling for selection into schools. The parent ADHD‐PGS has an effect of only β = −.002, reduced from −.037 without controlling for parents' education and income (Model 3). This suggests that the indirect genetic effect of parental ADHD‐PGS on offspring achievement is mediated by family socioeconomic status. Here, parental education has a stronger association with student achievement than parental income (0.25 vs. 0.09, respectively).

Note that results were similar for maths, reading and English outcomes (Table S5; Figure S5), and for boys and girls (Table S6).

**Figure 2:**
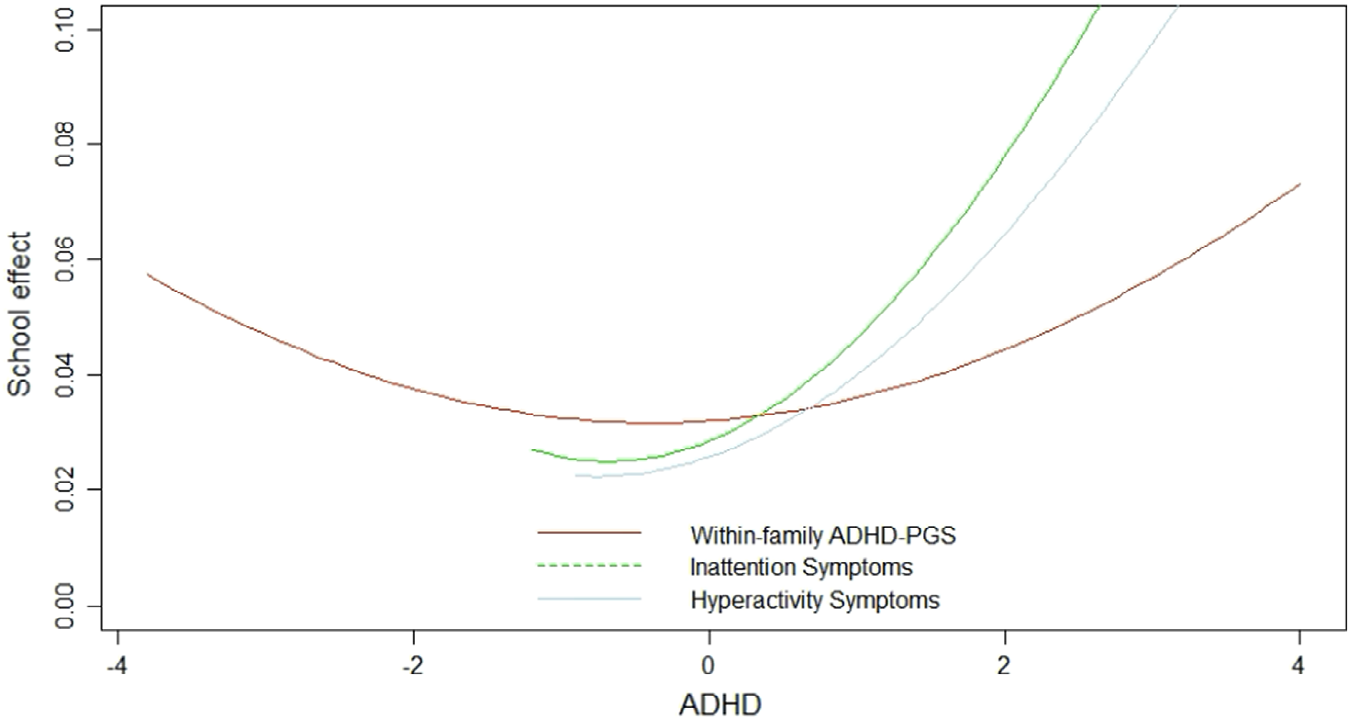
Variation in the effects of schools on achievement by student ADHD measures. On the *x*‐axis, 0 = mean level of each ADHD indicator, and values either side represent SDs from the mean, with lines covering only the range of observed variation. *Y*‐axis indicates the % variance explained in achievement by school, controlling for family background [Colour figure can be viewed at wileyonlinelibrary.com]

The ADHD–school interaction that we identified implies that not only do ADHD effects vary between schools but that school effects vary according to student ADHD levels. Figure 2, based on results from Model 4, shows the fraction of variance in achievement that is explained by school differences across ADHD levels. Schools are more important for students with higher compared to average ADHD symptoms and genetic risk. At average levels of ADHD inattention symptoms (i.e. *x*‐axis = 0 in Figure 2), differences between schools explain less than 2% of the variance in achievement. However, differences between schools become of greater importance when comparing students with increasingly high inattention symptoms; for those with the highest levels of symptoms, schools explain 10% of the variation in achievement. Nearly, the same estimates are found for hyperactivity symptoms. The pattern for ADHD‐PGS is similar; only the differences are smaller. Since parental income and education were controlled for, school effects reflect school ‘value added’, beyond the contribution of family background.

The correlation between the ADHD effect and the school effect on achievement, known as the slope–intercept correlation, was positive for all ADHD indicators (*r* = .36, .38 and .10 for inattention, hyperactivity and within‐family ADHD‐PGS, respectively). This positive correlation means that ADHD has less of an impact on achievement in higher‐achieving schools. Indeed, as ADHD effects increase between schools from strongly to weakly negative, overall achievement increases. As with the school intercept, the intercept–slope correlation is interpreted for average values of ADHD variables. We found that the correlation becomes more strongly positive with increasing values of student ADHD symptoms and PGS (Figure S6).

### The roles of school sociodemographic measures and special educational support

Our five direct measures of school sociodemographics contributed modestly to the between‐school differences in achievement. Model 5 showed improved fit relative to Model 4 (Table 2), but the school intraclass correlation – a latent school effect on achievement – reduced merely from 2.8% to 2.4% when including the school covariates. Effect sizes were β = 0.01 for school‐average parental education, 0.04 for inequality in parental education, 0.05 for average parental income, −0.05 for inequality in parental income and 0.06 for the proportion of non‐Western immigrants.

None of the five school‐level sociodemographic measures could explain ADHD‐by‐school interactions. This was reflected by the reduced fit of Model 6, which included ADHD‐by‐covariate interactions, relative to Model 5.

Supplementary analyses revealed tentative evidence that the relationship between ADHD and achievement depends on whether a student special educational support and that this dependency varies between schools (at least for inattention symptoms). This suggests that differential quality of special support between schools may explain our interaction results (see Supporting Information and Table S7).

## Discussion

Using a unique sample of ~23,000 Norwegian children with data on standardised national test results, school identifiers, ADHD symptoms and parent–offspring ADHD polygenic scores (PGS), we provide new insights into how achievement in this sample is shaped by the interplay between school contexts, and individuals' ADHD symptoms and genetic risk. The availability of parent–offspring ADHD‐PGS allowed us to take advantage of the ‘natural experiment’ of random segregation of parental genetic variation at conception. Using such a within‐family ADHD‐PGS design is equivalent to randomising children to schools, meaning that the genetic analyses (but not the symptom analyses) are unlikely to be confounded by selection into schools.

We detected an interaction between schools and ADHD, whether ADHD was measured by symptoms of inattention, hyperactivity or within‐family ADHD‐PGS. This indicates that the educational consequence of ADHD depends on which school ADHD is expressed in. For example, the average effect of inattention on achievement is β = −.23, but in the 2.5% of schools where inattention matters the least for achievement, the effect is β = −.07 or less. Our estimates of ADHD‐PGS associations with achievement (β = −.11 crude and −.07 within‐family) were similar to those from a prior study, although the proportion of the ADHD‐PGS association due to direct child‐led genetic effects was weaker for their Swedish GPA outcome (β = −.12 crude and −0.04 within‐family) (Jangmo et al., 2021). We build on this by showing that the within‐family ADHD‐PGS effect depends on the school, with effects greater than β = −.16 in the 2.5% of the schools with the most strongly negative slopes. The interaction also indicates that school effects on achievement vary according to students' levels of ADHD symptoms or genetic risk, with larger achievement differences between schools for higher ADHD students. For example, schools explain 2% of the variance in achievement for those with average inattention levels, versus 8% for students more than two standard deviations above average in inattention. Sociodemographic differences between schools cannot explain the latent ADHD‐by‐school interaction.

We found that in some schools, student achievement is strongly differentiated by ADHD, and in others, not at all. Our formal model comparisons indicated that the between‐school variation in ADHD effects is greater than expected by chance. For individual differences researchers, these school differences imply that associations between ADHD and achievement should be contextualised. For policymakers and teachers, this suggests that the link between ADHD and low achievement is malleable. On average, ADHD symptoms and genetic risk have large negative effects on achievement – with the strongest negative effects in the lower performing schools. However, this does not need to be the case. Further studies with richer data are needed to describe school contexts in which the ADHD penalty is reduced. Nevertheless, the finding of latent between‐school variation provides optimism by showing that schools where ADHD effects are small do exist.

The ADHD‐by‐school interaction also implies that school effects vary according to individual differences in ADHD symptoms and genetic risk. For those interested in education policy, this means that effects of school contexts must be interpreted with reference to individual psychological differences. The small impact of the school on national test scores on average (<3% of the variance) corresponds well with prior evidence from Norway and the United Kingdom (Hermansen, Borgen, & Mastekaasa, 2020; von Stumm et al., 2020). However, our results suggest that average estimates of school effects conceal larger impacts for certain children at risk of poor achievement. Students with high‐ADHD symptoms and genetic risk may achieve differently at different schools, even taking family background into account.

The greater impact of school differences on achievement differences among students with higher ADHD symptoms and genetic risk could reflect variability between schools in support for higher needs students. Special needs education has been shown to affect marginal participants' educational outcomes (Ballis & Heath, 2021), so variation in such practices could contribute to between‐school differences in ADHD effects. Norwegian national regulations emphasise inclusive education, with 92% of special needs students remaining in mainstream settings (Nes, 2017). However, schools differ in whether/how funds are allocated to higher needs students, and in how ‘inclusivity’ is interpreted and implemented. School psychology services are locally organised and may vary in the level of engagement and type of pedagogical interventions. Moreover, schools vary in how actively the principal facilitates adaptations of classroom practices and collaboration with school psychologists. Research suggests that many Norwegian schools do not follow the national policy of providing support as early as possible for learning difficulties, but instead require children to wait for an ADHD diagnosis (Haug, 2014). Our supplementary analyses provide initial evidence that schools vary in how much special educational support weakens the negative association between inattention and achievement. The role of special education in the ADHD–school interaction will be clarified upon the availability of richer school‐level data, or ideally data from experiments where children are randomised to receive special support. Future work should identify school environments that support high‐ADHD‐risk students to perform well. If a policy goal is to reduce school‐driven inequalities in achievement, efforts should focus on evening out differences in opportunities for students with high‐ADHD symptoms and genetic risk between schools.

We also tested whether available measures of school sociodemographics could account for the latent interactions between schools and students' ADHD symptoms and PGS. None of these factors (school‐average parental education, income, inequality in education, inequality in income, and proportion of non‐Western immigrants) appeared to be involved. These measures are likely too broad to capture how schools moderate the effect of ADHD on achievement. Future work should use the framework introduced here to identify the total latent moderation effect by schools, and then see what fraction of this can be captured by more detailed school environmental measures. Candidate school measures should include institutional, practical, social and community domains, for example, not only the qualifications of teachers and psychologists to cater to children with cognitive and behavioural difficulties (Taylor et al., 2010), but also time spent outdoors (potentially beneficial to students with tendencies for hyperactivity). Difficulties with measuring school indicators accurately (worse for measures such as school goals and leadership than for teacher qualifications and class size (Mayer, Mullens, & Moore, 2000)) add to the issue that we lack concrete statistical evidence on school factors that aid student achievement. Yet, it will be valuable to identify salient school features that aid the achievements of students with ADHD, since these may provide useful intervention targets. Future research should aim not only to explain the interaction identified within the current methodological framework but also to validate the finding through triangulation with other methods, such as regression discontinuity designs based on policy changes (Barcellos, Carvalho, & Turley, 2018).

This study has several limitations. First, the generalisability of the findings is limited. Analyses included a subsample of MoBa participants with European ancestries, as defined by principal component analysis of the genetic data. The findings cannot be uncritically generalised to children with non‐European ancestries, and replication of our findings in more diverse samples is essential. Generalisability issues also may stem from selection into the MoBa sample, and attrition linked to ADHD. However, the school identifiers and standardised national test results cover the Norwegian population and are not affected by these issues.

Second, while we control for passive gene–environment correlation using parental ADHD‐PGS, children's own genetic propensities could theoretically still influence their school attendance. However, this is unlikely for two reasons. First, the within‐family ADHD‐PGS shows no clustering in schools. Second, there are no selective elementary schools in Norway, with almost all children simply attending their local school. We note that self‐selection will be much more important in other educational settings (e.g. the United Kingdom), and will be difficult to account for even in within‐family gene–environment interaction studies. However, selection into schools is an interesting phenomenon that can be understood better using multilevel genetically sensitive methods.

Third, our analyses underestimate the main and interactive effects of ADHD genetic risk. The latest ADHD‐PGS explains only 5.5% of the variance in case–control ADHD (Demontis et al., 2017), despite the high twin heritability of 70–80% (M. A. Nikolas & Burt, 2010). Moreover, the PGS is based on mean effects of SNPs on ADHD case‐status across international education systems. These might not be the SNPs with effects that are sensitive to school context in Norwegian young people. However, the within‐family ADHD‐PGS had a strikingly similar average effect to hyperactivity symptoms (−0.07 and −0.09, respectively) and have the key advantage of providing a stricter test for gene–environment interaction. Indeed, compared with the measured ADHD symptoms, the ADHD‐PGS is less prone to the issue of selection into schools, more normally distributed and not subject to attrition. The interaction between genetic risk for ADHD and schools is likely to be larger than estimated here. We anticipate that future ADHD‐GWAS (ideally within‐family GWAS (Howe et al., 2021), capturing SNP effects on ADHD and *variability* in ADHD (Al Kawam, Alshawaqfeh, Cai, Serpedin, & Datta, 2018)) will facilitate more powerful PGS to be used in gene–environment interaction studies.

## Conclusion

In sum, we found evidence of an interaction between students' ADHD symptoms and PGS, and their schools. This means that the performance gap associated with ADHD differs between schools, and the impact of school on achievement varies according to individual differences in ADHD. Children with elevated ADHD symptoms and PGS perform better in some schools than others. Knowing the characteristics of these environments requires future research, but this study indicates that one can expect to find them. Given the high individual and societal costs of ADHD and low educational achievement (Du Rietz et al., 2020), the potential profitability of identifying these school qualities is great.

## Supporting information


**Appendix S1.** Sample size flowchart.
**Appendix S2.** Mobagenetics quality control.
**Appendix S3.** Analysis of special educational support data.
**Figures S1–S3**. Distributions of achievement by decile of average school performance.
**Figure S4.** Correlations between raw standardised test scores and item response theory‐derived scores.
**Figure S5.** Distributions of ADHD effects on achievement in individual subjects across Norwegian schools.
**Figure S6.** Slope intercept correlations (*y*‐axis) vary by ADHD levels (*x*‐axis).

**Table S1.** Intraclass correlations indicating variance in ADHD measures due to variation between schools.
**Table S2.** Phenotypic descriptive statistics for analysis variables within the MoBa genetics cohort.
**Table S3.** Fit statistics for main models (1–6).
**Table S4.** Results for main models (1–6).
**Table S5.** Results for maths, reading and english separately.
**Table S6.** Results for boys and girls separately.
**Table S7.** Results for special educational support analyses.
