# Supplementary material for: How interactions between ADHD and schools affect educational achievement: a family‐based genetically sensitive study
Source: J Child Psychol Psychiatry. 2022 Jul 4;63(10):1174–85. doi: 10.1111/jcpp.13656 (PMC9796390; doi:10.1111/jcpp.13656)
Supplement: Supplementary file 1 — Appendix S1. Sample size flowchart. Appendix S2. Mobagenetics quality control. Appendix S3. Analysis of special educational support data. Figures S1–S3. Distributions of achievement by decile of average school performance. Figure S4. Correlations between raw standardised test scores and item response theory‐derived scores. Figure S5. Distributions of ADHD effects on achievement in individual subjects across Norwegian schools. Figure S6. Slope intercept correlations (y‐axis) vary by ADHD levels (x‐axis). [file JCPP-63-1174-s002.docx]

**Supporting Information**

Cheesman et al. How interactions between ADHD and schools affect educational achievement: a family-based genetically sensitive study

Table of Contents

[Appendix S1 Sample size flowchart 2](#_Toc106290630)

[Appendix S2 Mobagenetics quality control 2](#_Toc106290631)

[Appendix S3 Analysis of special educational support data 2](#_Toc106290632)

[Figures S1- S3: distributions of achievement (average of maths, reading and English) by decile of average school performance, for grades 5, 8 and 9, separately. 5](#_Toc106290633)

[Figure S4: correlations between raw standardised test scores and item response theory-derived scores for MoBa participants. 8](#_Toc106290634)

[Figure S5: Distributions of ADHD effects on achievement in individual subjects across Norwegian schools. 9](#_Toc106290635)

[Figure S6: Slope intercept correlations (y-axis) vary by ADHD levels (x-axis). 10](#_Toc106290636)

## Appendix S1 Sample size flowchart

| **Step** | **Sample size** |
| --- | --- |
| MoBagenetics initial sample size 🡪 | 98110 individuals |
| Postimputation QC 🡪 | 93582 individuals; ~25,000 complete trios |
| Select one child per family with data on parent SES and school sociodemographics🡪 | 23598 complete trios for PGS analyses |
| Select children with age 8 ADHD symptom data 🡪 | 10718 children for symptom analyses |

## Appendix S2 Mobagenetics quality control

Quality control exclusion criteria for individuals were: genotyping call rate <95%, or autosomal heterozygosity >4 standard deviations from the sample mean. Quality control exclusion criteria for SNPs (single nucleotide polymorphisms) were: ambiguous (A / T and C / G), genotyping call rate <98%, minor allele frequency <1%, or Hardy-Weinberg equilibrium P-value <1 × 10-6. Population stratification was assessed, using the HapMap phase 3 release 3 as a reference, by principal component analysis using EIGENSTRAT version 6.1.4. Visual inspection identified a homogenous population and individuals of non-European ancestries were removed based on principal component analysis of markers overlapping with available HapMap markers. The parent and offspring datasets were then merged into one dataset per genotyping batch, keeping only the SNPs that passed quality control in both datasets. Phasing was conducted using Shapeit2 release 837 and the duoHMM approach was used to account for the pedigree structure. Imputation was conducted using the Haplotype reference consortium (HRC) release 1-1 as the genetic reference panel. The Sanger Imputation Server was used to perform the imputation with the Positional Burrows-Wheeler Transform (PBWT). The phasing and imputation were conducted separately for each genotyping batch. More detailed information about the cohorts, quality control and imputation can be found at <https://github.com/folkehelseinstituttet/mobagen>.

## Appendix S3 Analysis of special educational support data

We accessed parent-reported information from MoBa (at child age 8) on whether a formal administrative decision had been made about their child being eligible for special education. We created a binary variable with 1 indicating that the parent had reported ‘yes’ to the child receiving support in at least one of the following domains: Norwegian, arithmetic, ‘other subjects’, help for ‘a disability or developmental problem’. Of the 11,367 children with non-missing data for this variable, 7% had received special educational support for at least one school subject.

First, we excluded all children who had received support and re-ran Models 1-3 for all ADHD variables. If differences in achievements of children with special educational needs between schools drive our results, then the variability between schools in the effects of ADHD on achievement should be reduced when these individuals are excluded (i.e. comparing Model 3 before and after exclusions). The standard deviations of slopes for ADHD effects between schools reduced from 0.09 to 0.067 for inattention, to 0.079 to 0.66 for hyperactivity, and from 0.047 to 0.036 for the within-family ADHD-PGS (Table S6a). However, the remaining variability between schools in effects of all of the ADHD indicators on achievement among children without special needs status was still substantial, indicated by improved fit of Model 3 (upon inclusion of random slopes) compared to Model 2 (Table S6b).

To further investigate the finding that children with special educational needs play a role in the variability between schools in the impact of ADHD on achievement, we explicitly modelled the nature of this (rather than simply excluding the relevant children). We tested the fit of multilevel models allowing the effect of ADHD on achievement to vary according to whether special education was received (i.e., including an ADHD*special education interaction as a fixed effect) and then allowing for this interaction to differ between schools (including the interaction as a random slope effect). Improved fit of the model upon inclusion of the ADHD*special education random slope would indicate that special educational support changes the ADHD-achievement association to different extents in different schools.

We found that the interaction between the effect of inattention and the effect of being a special education student on achievement differed between schools (model 10 was supported; Table S6b). The average effect size of the interaction was positive, meaning that on average, students with special educational needs show a weaker association between inattention and achievement (less strongly negative). This is in line with our prior evidence that higher-performing schools (a positive environment, like having special educational support) have weaker ADHD-achievement associations. The correlation between school intercepts and slopes for the interaction was -0.32. In lower performing schools, the interaction of special education and the inattention-achievement association is therefore more positive. This indicates that the inattention-achievement association is stronger in lower-achieving schools for those who don't receive support. In higher-achieving schools, those receiving support have a weaker negative inattention-achievement association that is more similar to the association for those not receiving support. We speculate that this pattern of findings is partly explained by students with low inattention having other neurodevelopmental difficulties, and being more differentiated from their non-special-education peers in achievement in lower-performing schools.

For hyperactivity and the within-family ADHD-PGS, model 9 was best-fitting, meaning that there was an interaction with special education status, but this interaction did not vary between schools.

All in all, this is inconclusive initial evidence that schools vary in how much their special needs support weakens the negative association between ADHD and achievement.

Multiple limitations of the current data make us reluctant to draw strong conclusions. First, interpretation is difficult because children who were already struggling academically were selected into special education (the outcome causes the exposure). Second, we have no information on the quality of special education. Third, our special education variable was reported at child age 8, so may miss children who received support later on yet before their standardised tests were taken. Fourth, we only have data for the special education status of MoBa participants, and so cannot create representative school-aggregate measures (i.e., whereas for the register-based school sociodemographic measures we could characterise school-average parental income, here we cannot calculate the fraction of children in each school who receive special support). Fifth, it is likely that only the children with the highest symptoms/PGS were receiving special support specifically for ADHD. Children receiving special support who have low ADHD symptoms might nevertheless be struggling with severe learning disabilities and are therefore not very comparable to children with similar ADHD symptoms but who are not receiving support. A more informative, and less stringent test could be to examine school differences in how ADHD-specific support (rather than for all support including e.g., dyslexia) changes the ADHD-achievement association. Finally, missingness for the special education variable makes the results difficult to compare to the original study results – particularly for the polygenic score analyses, which were originally not dependent on the presence of MoBa phenotype data.

The role of special education in the ADHD-school interaction will be clarified upon the availability of richer school-level data, or ideally data from experiments where children were randomised to receive special support.

## Figures S1- S3: distributions of achievement (average of maths, reading and English) by decile of average school performance, for grades 5, 8 and 9, separately.

The histograms demonstrate the shift in mean performance with increasing school intercepts. Achievement data are not skewed (skewness <-1 or >+1) within any of the school intercept deciles, and there is substantial variability within each decile.


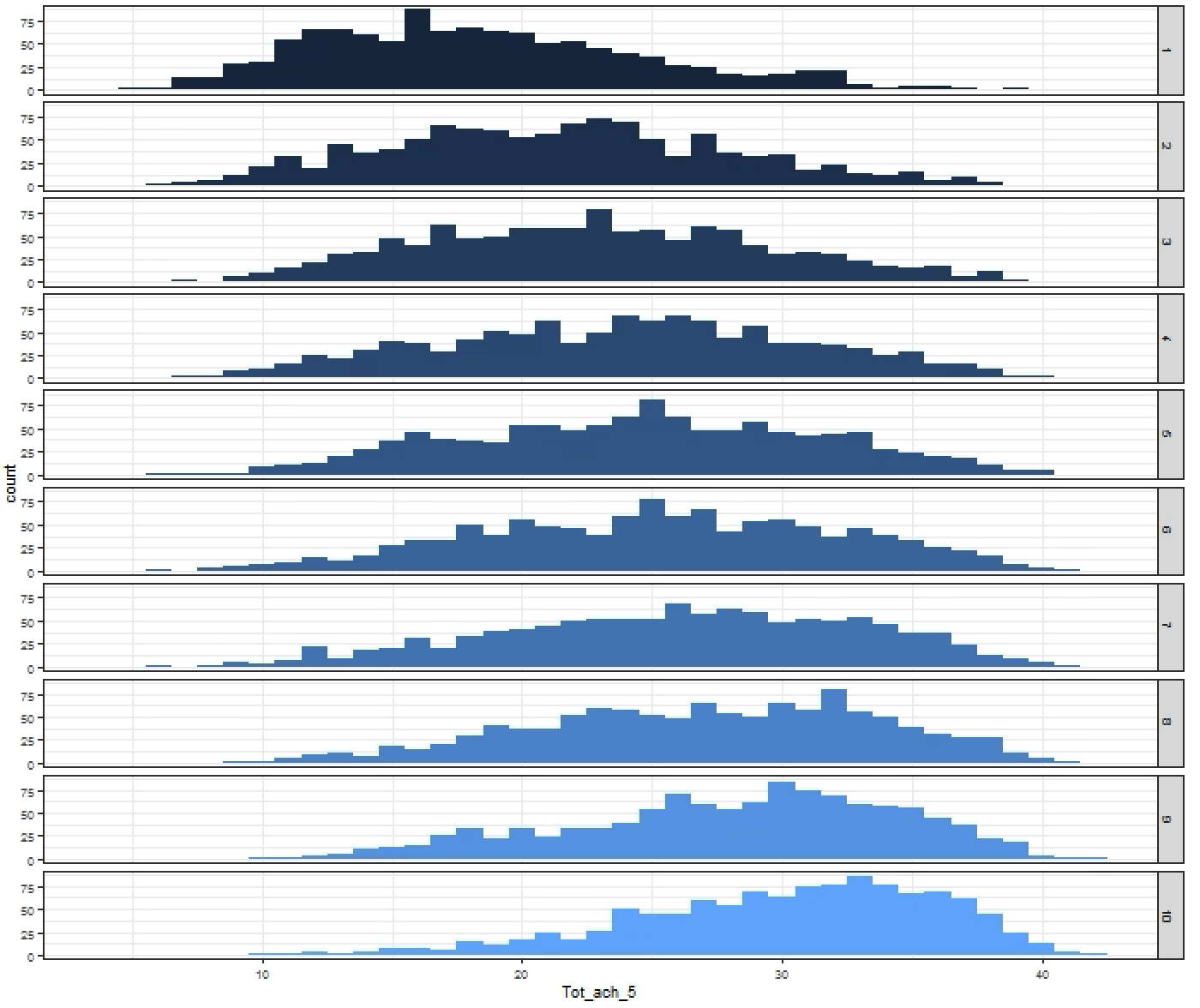


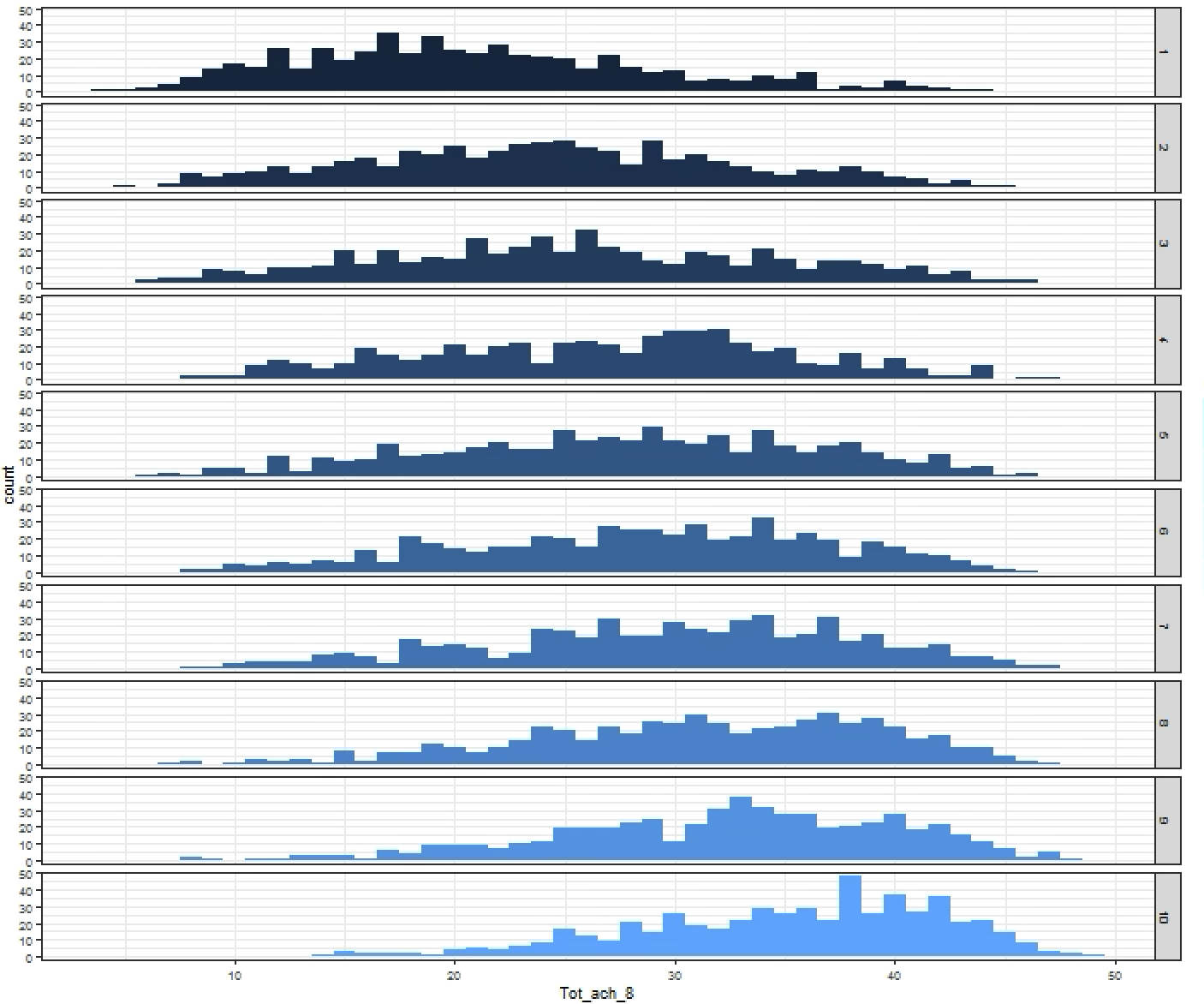


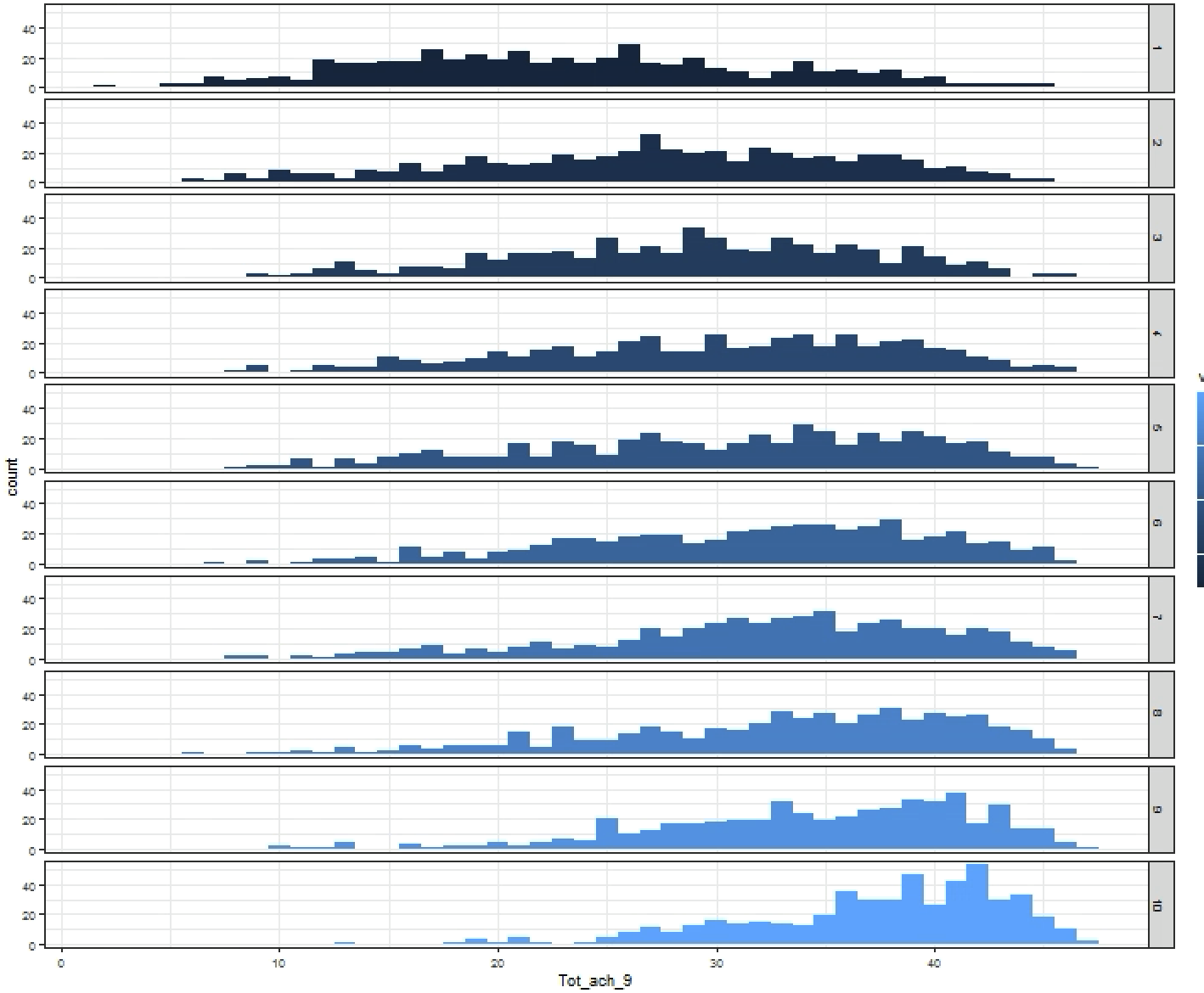


Figure S4: correlations between raw standardised test scores and item response theory-derived scores for MoBa participants.

Note: POENG=points, SKALAPOENG= IRT scores, R=reading, M=maths, E=English, 5/8/9= grades. Correlations ranged from 0.982 (for reading grade 9) to 0.993 (maths grade 9).


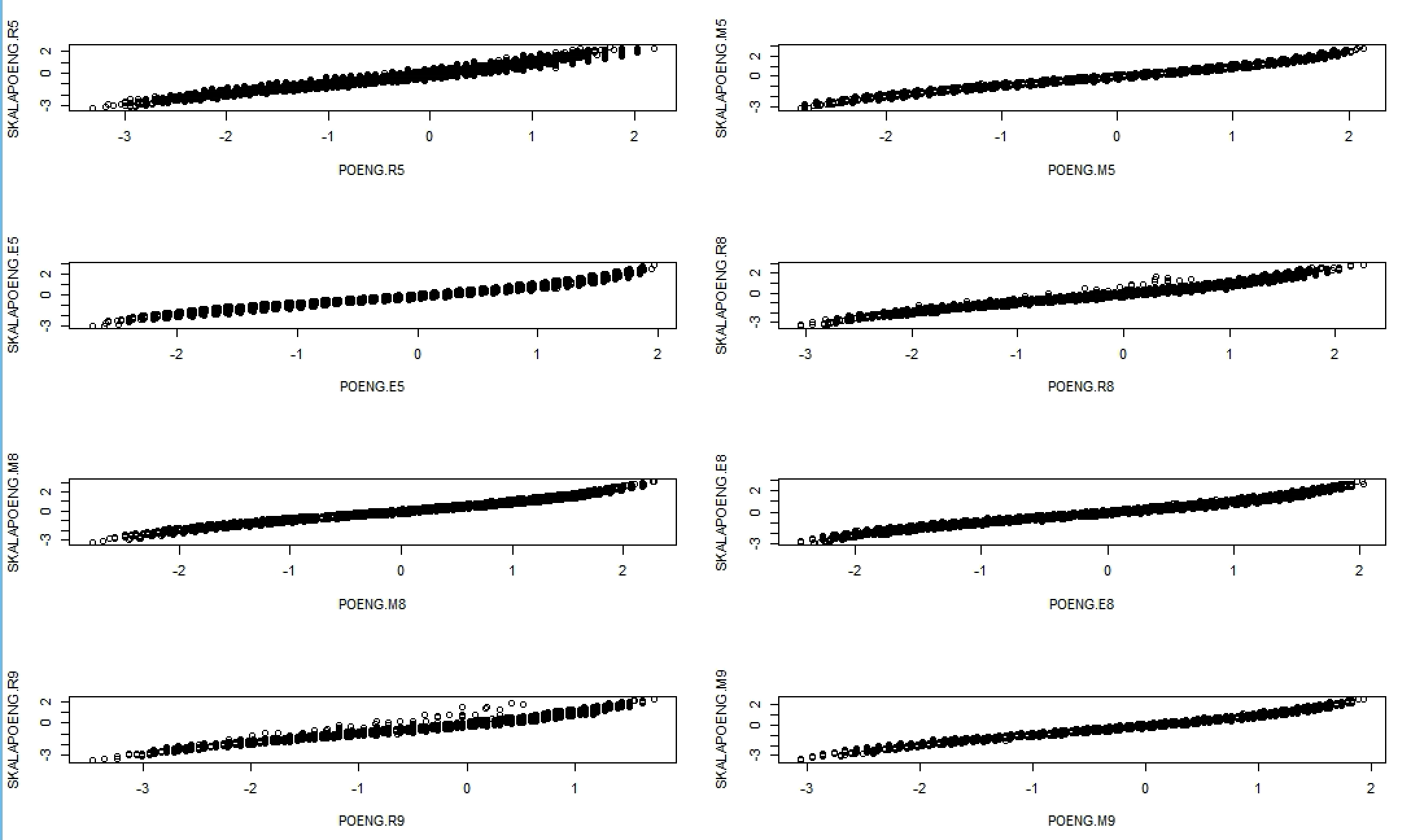


## Figure S5: Distributions of ADHD effects on achievement in individual subjects across Norwegian schools.

Note: The overall pattern of results was similar between subjects in terms of average effects and variability between schools. The within-family ADHD-PGS had almost identical effects on all outcomes. Inattention had stronger effects on maths than reading. There is no red curve for English because the model including different ADHD-PGS effects between schools did not fit the data better than the school intercept-only model.

**
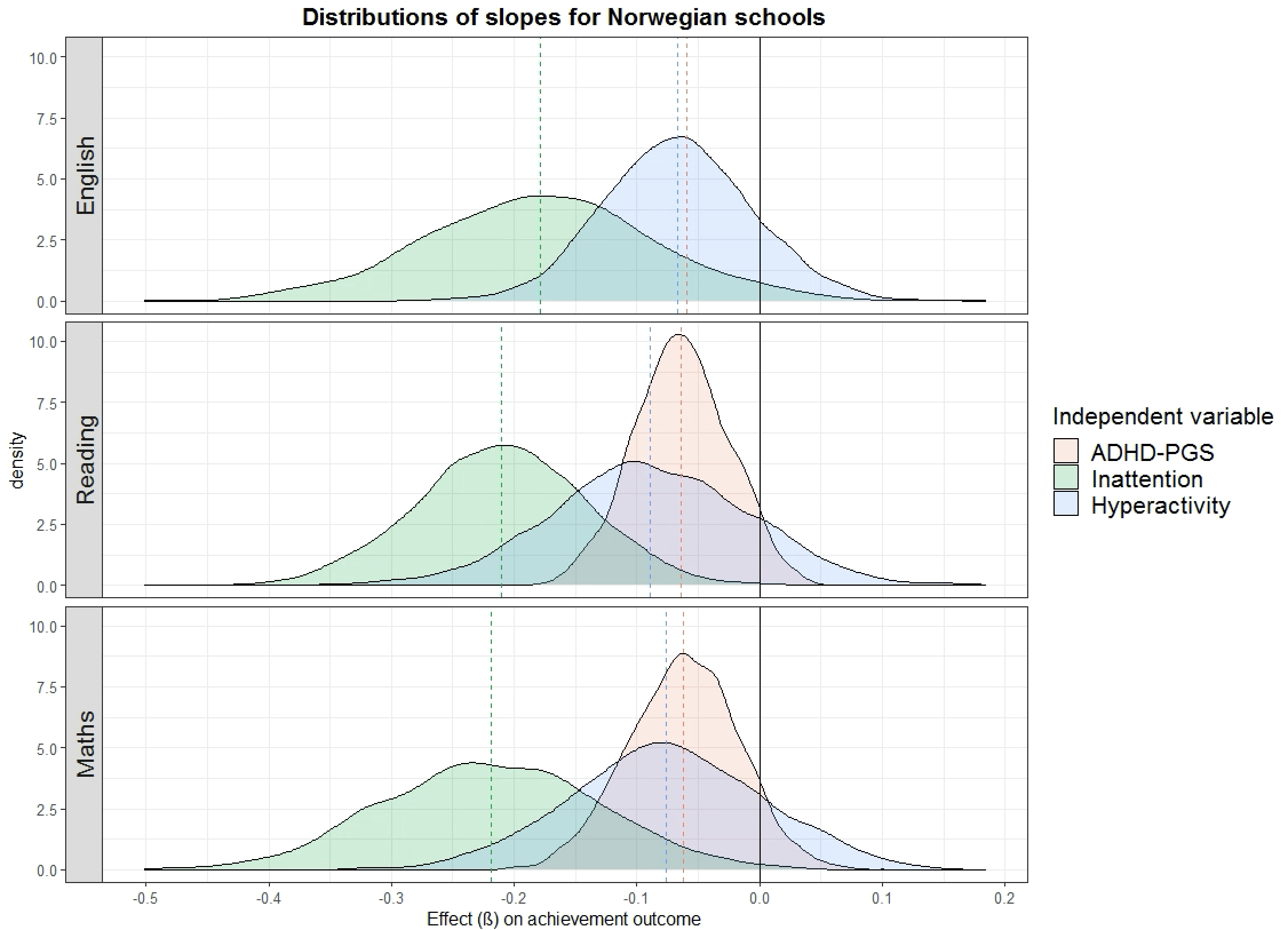
**

## Figure S6: Slope intercept correlations (y-axis) vary by ADHD levels (x-axis).

**
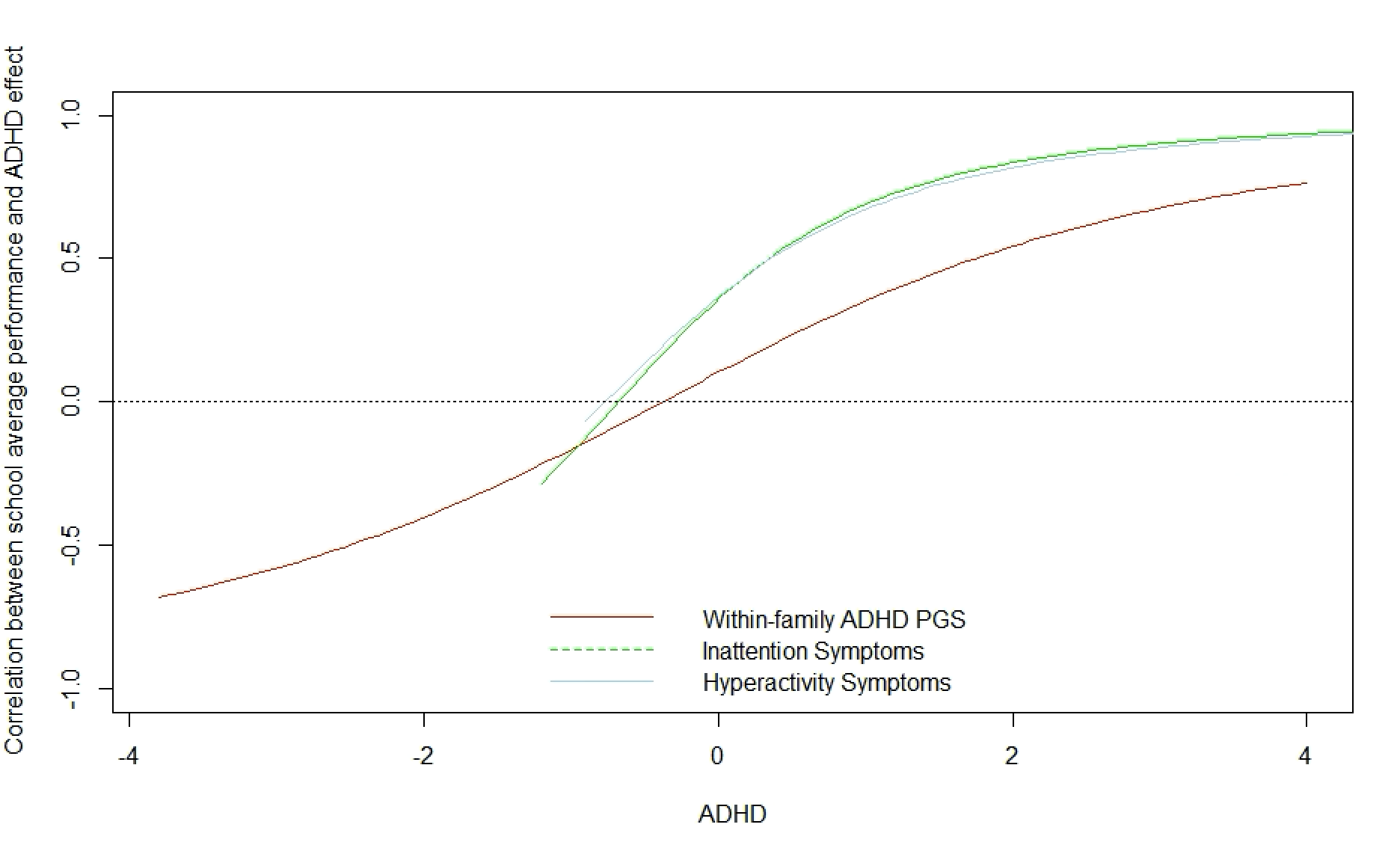
**
